# Synthesis, Characterization, Antimicrobial Screening and Free-Radical Scavenging Activity of Some Novel Substituted Pyrazoles

**DOI:** 10.3390/molecules200610468

**Published:** 2015-06-08

**Authors:** Nagwa Mohamed Mahrous Hamada, Nadia Yousef Megally Abdo

**Affiliations:** Department of Chemistry, Faculty of Education, Alexandria University, Alexandria 21526, Egypt; E-Mail: nadiamegally@yahoo.com

**Keywords:** chalcones, sulfonamide pyrazole, isonicotinic acid hydrazide, antimicrobial activity, antioxidant, anti-inflammatory

## Abstract

The present work deals with the synthesis of acetoxysulfonamide pyrazole derivatives, substituted 4,5-dihydropyrazole-1-carbothioamide and 4,5-dihydropyrazole-1-isonicotinoyl derivatives starting from substituted vanillin chalcones. Acetoxysulfonamide pyrazole derivatives were prepared from the reaction of chalcones with *p*-sulfamylphenylhydrazine followed by treatment with acetic anhydride. At the same time 4,5-dihydropyrazole-1-carbothioamide and 4,5-dihydropyrazole-1-isonicotinoyl derivatives were prepared from the reaction of chalcones with either thiosemicarbazide or isonicotinic acid hydrazide, respectively. The synthesized compounds were structurally characterized on the basis of IR, ^1^H-NMR, ^13^C-NMR spectral data and microanalyses. All of the newly isolated compounds were tested for their antimicrobial activities. The antimicrobial screening using the agar well-diffusion method revealed that the chloro derivatives are the most active ones. Moreover, the antioxidant and anti-inflammatory activity of these chloro derivatives are also studied using the DPPH radical scavenging and NO radical scavenging methods, respectively.

## 1. Introduction

**Figure 1:**
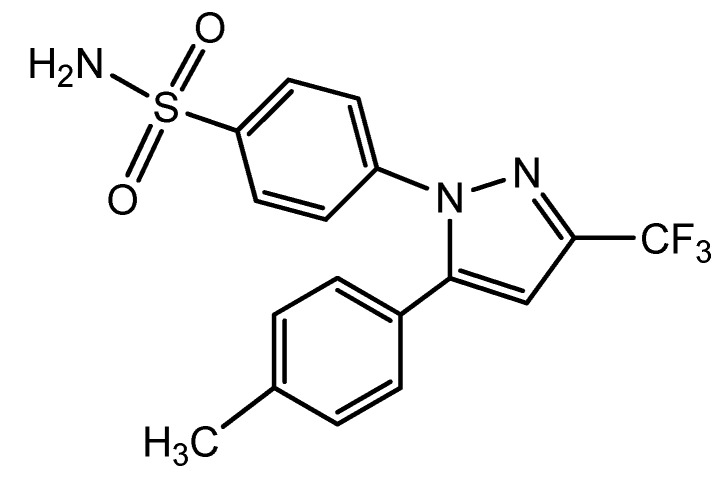
Structural formula of celecoxib.

Pyrazole and its derivatives represent one of the most active classes of heterocyclic compounds possessing a wide spectrum of biological activities. In particular, they are used as antitumor [1], antibacterial, antifungal, antiviral, antiparasitic, antitubercular, insecticidal, anti-inflammatory, antidiabetic and analgesic compounds [2,3,4,5,6,7,8,9,10,11,12,13,14]. Pyrazoles are also used extensively as useful synthons in organic synthesis [15,16,17,18,19,20,21,22]. A literature survey reveals that a significant portion of research in heterocyclic chemistry has been devoted to pyrazoles containing different aryl groups as substituents [23,24,25,26,27,28,29,30]. For example, celecoxib (Figure 1) is a sulfonamide non-steroidal anti-inflammatory drug [31]. The structure activity relationships of celecoxib attracted our attention and prompted us to synthesize some matching pyrazole derivatives with some structural modifications.

Similarly, there has been a considerable interest in the chemistry of the 2-pyrazoline ring system which is a core structure in various synthetic pharmaceuticals with a broad spectrum of biological activities. Their pharmaceutical importance lies in the fact that they can be effectively utilized as antibacterial, potential antimicrobial [32], anti-inflammatory [33], analgesic [34], antidepressant [35,36,37], anticancer [38], antiproteolytic [39], antiviral [40], antihypertensive [41], antitubercular [42], and anticonvulsant [43] compounds. As a part of our program aiming at the synthesis of different heterocyclic derivatives, we report here in the convenient synthesis of some new pyrazoles **3a**–**e** and dihydropyrazoles **4a**–**e** and **5a**–**e** starting from chalcones **1a**–**e**. which exhibit efficient antimicrobial, antioxidant and anti-inflammatory activities.

## 2. Results and Discussion

### 2.1. Chemistry

The synthesis of chalcones **1a**–**e** was accomplished by a one-pot Claisen-Schmidt condensation [44,45] in 60% ethanol between the appropriate acetophenone derivative and 4-hydroxy-3-methoxy-benzaldehyde (vanillin). In all the synthesized chalcones, only the *trans* double bond was obtained (on the basis of the corresponding NMR coupling constant). All synthesized compounds were characterized by spectral data (IR, ^1^H-NMR and ^13^C-NMR) that was consistent with the proposed structures. The IR spectra of **1a**–**e** revealed the characteristic bands for C=O at 1661–1684, vinyl CH=CH that appeared at 1593–1618 and OH at 3424–3441 cm^−1^. The ^1^H-NMR spectra showed the presence of a broad singlet at δ = 11.22–11.72 ppm for the phenolic OH proton, multiplets at δ = 7.30–7.97 characteristic of the aromatic protons, a doublet (*J* = 15 Hz) at δ = 7.22–7.29 ppm characteristic of the olefinic COC*H*=CH, another doublet (*J* = 15 Hz) at δ = 6.61–6.89 ppm characteristic of the olefinic COCH=C*H*. The methyl protons appeared as a singlet in the δ = 3.21–3.36 ppm range.

The hydrazone derivatives **2a**–**e** were obtained by treatment of **1a**–**e** with *p*-sulphamylphenyl- hydrazine in glacial acetic acid. The IR spectra of **2a**–**e** showed the characteristic bands for a vinyl CH=CH group at 1603–1619 cm^−1^, the phenolic OH in the 3430–3441 cm^−1^ range and a primary or secondary amine band at 3379–3391 and 3310–3350 cm^−1^, respectively. The ^1^H-NMR spectra showed the presence of a singlet at δ = 11.22–11.39 ppm for the OH proton, a singlet equivalent to one proton in the δ = 8.21–8.73 ppm range characteristic of a hydrazone NH proton, while the primary amine NH_2_ protons appeared at δ = 9.23–9.40 ppm. A multiplet at δ = 7.26–7.82 ppm is characteristic of the aromatic protons, while a doublet at δ = 7.53–7.61 ppm for N=C–C*H*=CH (*J* = 13 Hz), another doublet at δ = 6.69–6.85 ppm (*J* = 13 Hz) for N=C–CH=C*H* and a singlet equivalent to three protons at δ = 3.19–3.37 ppm are characteristic of the CH_3_ protons, respectively.

Reaction of **2a**–**e** with acetic anhydride produced the pyrazole acetate derivatives **3a**–**e** in good yields. The structures of **3a**–**e** was confirmed by their IR, ^1^H-NMR and ^13^C-NMR spectra. The IR spectra of **3a**–**e** showed the characteristic bands for C=N at 1632–1656 cm^−1^, ester carbonyl band at 1746–1752 cm^−1^and the NH_2_ band appeared at 3379–3393 and 3250–3330 cm^−1^. Also, the ^1^H-NMR spectra of **3a**–**e** revealed the following signals: a singlet equivalent to two protons at δ = 9.33–10.37 ppm characteristic of NH_2_ protons, a multiplet at δ = 7.13–8.29 ppm characteristic for the aromatic protons, a singlet for the pyrazole C_4_-H at δ = 6.72–6.93 ppm, beside the presence of two singlets at δ = 2.22–2.39 ppm and δ = 3.21–3.33 ppm corresponding to the methyl protons of OCOCH_3_ and OCH_3_, respectively. Moreover, the ^13^C-NMR spectrum of **3d**, as an example of this series, showed different characteristic signals at δ 20.7 (CH_3_), 56.0 (OCH_3_), 168.8 (C=N) and 189.3 (C=O).

**Scheme 1:**
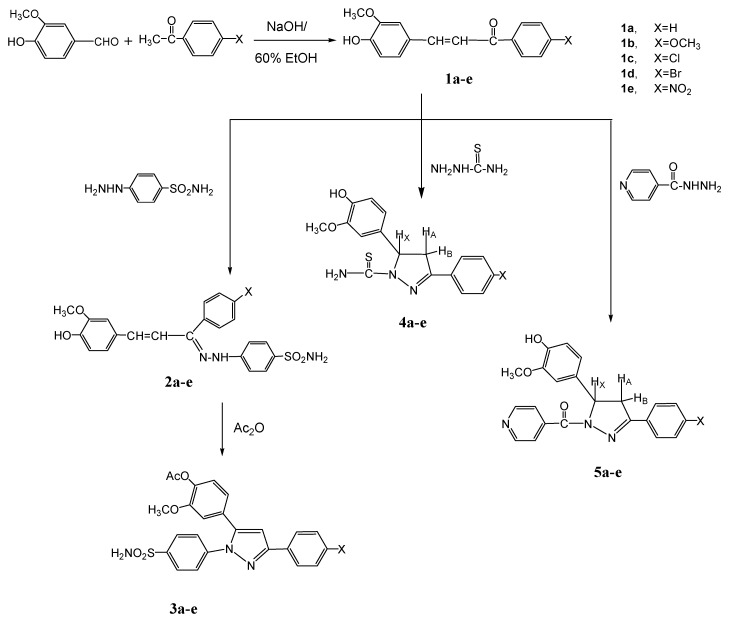
Synthesis of some novel substituted pyrazoles.

Condensation of chalcones **1a**–**e** with either thiosemicarbazide or isonicotinic acid hydrazide in ethanol containing a few drops of acetic acid afforded the 4,5-dihydropyrazole derivatives **4a**–**e** and **5a**–**e**, respectively. The key reactions involved the intermediate formation of the hydrazones and subsequent addition of N-H on the olefinic bond of the propenone moiety that forms the ring-closed final products. The spectral data (IR, ^1^H-NMR and ^13^C-NMR) of **4a**–**e** and **5a**–**e** were in full agreement with the proposed structures. The absence of the carbonyl (CO) and olefinic (C=C) bands in the IR spectra of the 4,5-dihydropyrazole derivatives **4a**–**e** and **5a**–**e** proved the ring closure of the final products. The IR spectra of **4a**–**e** showed thiocarbonyl C=S stretching bands at 1232–1248 cm^−1^ and NH_2_ absorption bands at 3387–3394 cm^−1^, whereas the IR spectra of **5a**–**e** showed an amide carbonyl stretching band at 1628–1634 cm^−1^. Also, the ^1^H-NMR spectra of either **4a**–**e** or **5a**–**e**, revealed the presence of a pair of doublets of doublets corresponding to the ring protons (H_A_ and H_B_) of 4,5-dihydropyrazole. The CH protons (H_X_) appeared as doublets of doublets due to vicinal coupling with the two magnetically non-equivalent protons of the methylene group H_A_ (up field shift of CH_2_) and H_B_ (downfield shift of CH_2_) at position 4 of the dihydropyrazole ring (*J*_AB_ = 16 Hz, *J*_AX_ = 3.6 Hz, *J*_BX_ = 12 Hz). Moreover, the NH_2_ protons of the thiocarbamoyl group of the dihydropyrazoles **4a**–**e** appeared at δ = 10.71–10.93 ppm, generally as broad bands. At the same time the ^13^C-NMR spectrum of **4b** showed different signals at δ: 48.2, 70.6 (C_3_ and C_4_ dihydropyrazole), 56.2, 56.5 (2 OCH_3_), 169.4 (C=N), 177.8 (C=S) and that for **5c** revealed the signals at δ: 40.3, 66.8 (C_3_ and C_4_ dihydropyrazole), 56.3 (OCH_3_), 164.3 (C=N), 188.4 (C=O). All the results are shown in Scheme 1.

### 2.2. Pharmacological Activity

#### 2.2.1. *In Vitro* Antibacterial Screening of Synthesized Compounds

All of our synthesized compounds, chalcones **1a**–**e**, hydrazones **2a**–**e**, pyrazoles **3a**–**e**, and dihydropyrazoles **4a**–**e** and **5a**–**e** were tested for their antimicrobial activity against four test organisms, namely *Staphylococcus aureus* ATCC6538P, *Escherichia coli* ATCC8739, *Pseudomonas aeruginosa* ATCC9027, and *Candida albicans* ATCC2091 using rifampicin (5 μg/disc) and ampicillin (10 μg/disc) as standard drugs. The agar well-diffusion method [46] was used for studying the potential activities of these compounds. Pyrazoles **3a**–**e** showed no significant effect, whereas all other compounds showed potent activity only against *Staphylococcus aureus* and *Candida albicans* in the following order: **2a**–**e** > **4a**–**e** > **1a**–**e** ≥ **5a**–**e**. Minimum inhibitory concentration (MIC) values for the individual compounds that showed inhibition zones > 10 mm were determined by means of the agar well-diffusion method in DMSO. The trend of activity was observed as follows: X > H > OMe > NO_2_ where X = Cl, Br. It is obvious that the presence of pharmacophores such as chloro and bromo substituents with lipophilic properties increases the antimicrobial activity. The activity results of our synthesized compounds against *S. aureus*, *C. albicans* are shown in Table 1 as zone of inhibition (in mm) and minimum inhibitory concentration, MIC (mg/mL).

**Table 1:** Determination of zone of inhibition and minimum inhibitory concentrations (MIC).

Compound No.	Zone of Inhibition (mm)		Minimum Inhibitory Concentration (MIC) mg/mL	
	*S. aureus*	*C. albicans*	*S. aureus*	*C. albicans*
**1a**	-	15	-	-
**1b**	-	15	-	-
**1c**	21	20	0.1	0.05
**1d**	15	18	0.063	0.063
**1e**	-	15	-	-
**2a**	19	22	0.063	0.031
**2b**	18	25	0.125	0.031
**2c**	22	26	0.05	0.05
**2d**	18	20	0.063	0.125
**2e**	17	17	-	-
**4a**	-	15	-	-
**4b**	-	15	-	-
**4c**	21	24	0.05	0.05
**4d**	-	15	-	-
**4e**	17	17	-	-
**5a**	-	20	-	0.25
**5b**	-	15	-	-
**5c**	17	20	0.1	0.05
**5d**	14	20	0.12	0.5
**5e**	12	15	-	-
Rifampicin	32	-	-	-
Ampicillin	30	-	-	-
DMSO	-	14	-	-

-: No activity.

Also, minimum bactericidal concentrations (MBC) were determined for all the chloro derivatives **1c**, **2c**, **4c** and **5c** which exhibited high activities. These results were listed in Table 2.

**Table 2:** Determination of minimum bactericidal concentration (MBC) of the chloro series.

Concentrations mg/mL	1	0.50	0.25	0.125	0.063	0.031	1	0.50	0.25	0.125	0.063	0.031
Microorganism Growth	*S. aureus*						*C. albicans*					
**1c**	−	−	*	+	+	+	−	*	+	+	+	+
**2c**	−	−	*	+	+	+	−	−	*	+	+	+
**4c**	−	*	+	+	+	+	−	−	−	*	+	+
**5c**	−	−	*	+	+	+	−	−	−	*	+	+

−: No turbidity; +: Turbidity; *: MBC

#### 2.2.2. Evaluation of Antioxidant and Anti-inflammatory Activities

Two pharmacological activities, namely antioxidant and anti-inflammatory activities, were tested for the chloro derivatives **1c**, **2c**, **3c**, **4c**, **5c**. These activities vary according to their structures and functional groups.

##### Antioxidant Activity (DPPH Based Free Radical Scavenging Activity)

The 1,1-diphenyl-2-picrylhydrazyl radical (DPPH) has been widely used to evaluate the free radical scavenging capacity of different antioxidants [47,48,49,50].

**Scheme 2:**
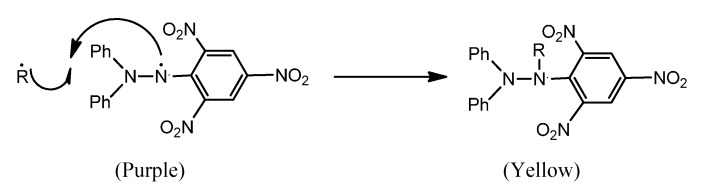
Reactions of DPPH.

Resulting from a color change from purple to yellow, the absorbance decreased when the DPPH is scavenged by an antioxidant, through donation of hydrogen to form a stable DPPH molecule, in the radical form this molecule had an absorbance at 517 nm, which disappeared after acceptance of an electron or hydrogen radical from an antioxidant compound to form the reduced DPPH-R (Scheme 2).

Moreover antioxidants are known to interrupt the free-radical chain of oxidation and to donate hydrogen from phenolic hydroxy groups, thereby, forming stable free radicals, which do not initiate further oxidation [51]. Furthermore, substitution in the aromatic ring system with halogens like chlorine or bromine sharply enhanced the antioxidant potency [52], it is thought that the chlorine atom because of its lone pair electron as well as its electronegative power enhanced the formation and subsequent stabilization of the nitrogen-ring radical through intervening aromatic system property, it might have enhanced the power to absorb free radicals, especially reactive oxygen and reactive nitrogen species (ROS and RNS), this is explain why we select this series of our synthesized compounds. The present investigation emphasized mainly on the chloro derivatives which showed significant antioxidant activity, the screening of the selected synthesized compounds through structure-activity relationship (SAR) showed that compound **2c** was found to be the most efficacious antioxidant among all the listed compounds. The antioxidant activity of **2c** is directly proportional to the concentrations used. Antioxidant results of the synthesized compounds **1c**, **2c**, **3c**, **4c** and **5c** are reported in Table 3. As reported in literature some substances can serve as either antioxidants or pro-oxidants, depending on conditions [53,54]. All the other tested compounds act as antioxidants at low concentrations (0.25 mg/mL) in the following order: **4c** > **5c** > **3c** > **1c**, while converted to pro-oxidant compounds at higher concentrations.

##### Anti-Inflammatory Activity (Scavenging of Nitric Oxide Radical)

Nitric oxide (NO) is a potent inhibitor of physiological processes such as smooth muscle relaxation, neuronal signaling, and inhibition of platelet aggregation and regulation of cell mediated toxicity [55]. In addition to reactive oxygen species, nitric oxide is also implicated in inflammation, cancer and other pathological conditions [56,57]. NO is known to be a ubiquitous free-radical moiety, which is distributed in tissues or organ systems and is supposed to have a vital role in neuromodulation or as a neurotransmitter in the CNS [58]. In our study all the chloro derivatives of the synthesized compounds **1c**, **2c**, **3c**, **4c**, **5c** were tested for *in vitro* anti-inflammatory activity compared to the standard vitamin C, showing acceptable anti-inflammatory activity. All tested compounds act as anti-inflammatory in a concentration dependent matter. Among all the tested compounds, **4c** was the most potent compound, followed by: **5c** > **3c** > **1c** > **2c**. The *in vitro* anti-inflammatory activity of tested compounds is summarized in Table 4.

**Table 3:** *In vitro* antioxidant activity data (DPPH scavenging). 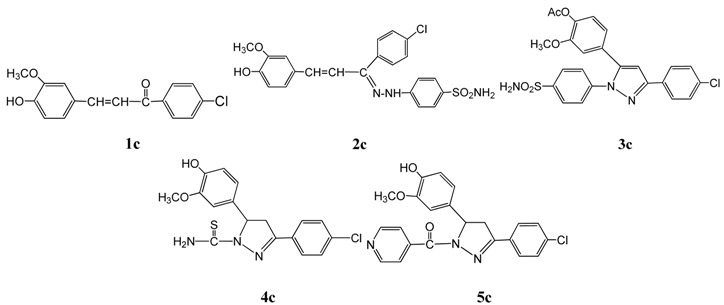

Compound No.	Mean Absorbance ± S.D.* at Different Concentrations				Efficacy at 0.25/0.25 Vitamin C
	0.25 mg/mL	0.5 mg/mL	0.75 mg/mL	1 mg/mL	
**1c**	9.4 ± 0.5	−65 ± 3.5	−69 ± 5.2	−88 ± 3.5	0.13
**2c**	58 ± 3.2	60 ± 2.5	91 ± 4.2	94 ± 6.5	0.81
**3c**	20 ± 2.4	12 ± 1.2	−12 ± 1.2	−5 ± 0.5	0.28
**4c**	78.8 ± 1.2	43.5 ± 2.5	31.8 ± 3.1	−69 ± 6	1.1
**5c**	64.7 ± 4.3	51 ± 2.3	21 ± 2.5	−7 ± 0.8	0.9
Vitamin C	72 ± 2.5	76 ± 1.8	89 ± 2.1	94 ± 2.8	

* S.D. = Standard deviation (Average of three determination).

**Table 4:** *In vitro* anti-inflammatory activity data (NO scavenging).

Compound No.	Mean Absorbance ± S.D.* at Different Concentrations				Efficacy at 0.25/0.25 Vitamin C
	0.25 mg/mL	0.5 mg/mL	0.75 mg/mL	1 mg/mL	
**1c**	36 ± 1.3	38 ± 2.1	41 ± 4.1	44 ± 1.5	0.88
**2c**	17 ± 1.7	19 ± 1.2	25 ± 1.5	29 ± 1.8	0.4
**3c**	7 ± 1.1	14 ± 1.2	25 ± 1.5	47 ± 3.5	0.94
**4c**	19 ± 1.9	36 ± 3.2	58 ± 2.4	74 ± 4.5	1.5
**5c**	26 ± 1.9	34 ± 1.5	42 ± 2.5	62 ± 5.2	1.03
Vitamin C				50 ± 1.2	

* S.D. = Standard deviation (Average of three determination).

## 3. Experimental Section

### 3.1. General Information

Melting points were determined in open capillary tubes using Electrothermal apparatus 9100 (Fisher Scientific, Leicestershire, UK) and are uncorrected. Microanalyses were operated at Faculty of Science, Cairo University, Cairo, Egypt, using an Elementary Vario El III C, H, N, S Analyzer (Shimadzu, Berlin, Germany). IR spectra were recorded using the potassium bromide method on a Tensor 37 FT-IR spectrometer (Bruker, Ettlingen, Germany): and expressed in wave number (υ_max_) cm^−1^. ^1^H-NMR and ^13^C-NMR spectra were measured in deuterated chloroform (CDCl_3_) or deuterated dimethyl sulphoxide (DMSO-*d*_6_) on an EAC 500 MHz FT-NMR spectrophotometer (Jeol, Eching, Germany). Chemical Shifts were recorded in δ as parts per million (ppm) downfield from tetramethylsilane (TMS) used as internal standard. Reaction progress and compound purity were monitored by Thin Layer Chromatography (TLC) using Alugram Sil G/UV_254_ silica gel plates (Macherey-Nagel, Easlon, PA, USA) and chloroform or chloroform-ethanol (9:1) or (19:1) as eluent systems. The spots were visualized using an ultraviolet lamp (Vilber Lourmet, Marine La Vallee, France) at λ = 254 and 266 nm. The antimicrobial activities were determined at the Department of Pharmaceutical Microbiology, Faculty of Pharmacy, Alexandria University. Antioxidant and anti-inflammatory activity tests were performed at the Biochemistry Lab, Faculty of Science, Alexandria University.

#### 3.1.1. General Procedure for the Preparation of **1a**–**e**

The appropriate *p*-substituted acetophenone (10 mmol) was added to a cold stirred solution of sodium hydroxide (3 g in 50 mL of 60%aqueous ethanol) followed by addition of vanillin (10 mmol) dropwise with continuous stirring for five hours. The resulting crude solid was filtered, washed successively with water, dried and crystallized from ethanol (95%) [44,45,59,60,61].

*3-(4-Hydroxy-3-methoxyphenyl)-1-(phenyl)prop-2-en-1-one* (**1a**). The chalcone was obtained in 73% yield; m.p. 93 °C; IR (KBr, cm^−1^): 3426 (OH), 1666 (C=O), CH=CH (1593); ^1^H-NMR (DMSO-*d*_6_): δ 3.35 (s, 3H, OCH_3_), 6.88 (d, 1H, COCH=C*H*, *J* = 15 Hz) , 7.26 (d, 1H, COC*H*=CH, *J* = 15 Hz), 7.34–7.86 (m, 8H, Ar-H), 11.32 (s, 1H, OH, D_2_O exchangeable); Anal. Calcd. for C_16_H_14_O_3_: C, 75.57; H, 5.55. Found: C, 75.42; H, 5.97.

*3-(4-Hydroxy-3-methoxyphenyl)-1-(4-methoxyphenyl) prop-2-en-1-one* (**1b**). The chalcone was obtained in 68% yield; m.p. 117 °C; IR (KBr, cm^−1^): 3441 (OH), 1661 (C=O), CH=CH (1502); ^1^H-NMR (DMSO-*d*_6_): δ 3.21 (s, 3H, OCH_3_), 3.33 (s, 3H, OCH_3_), 6.72 (d, 1H, COCH=C*H*, *J* = 15 Hz), 7.25 (d, 1H, COC*H*=CH, *J* = 15 Hz), 7.32–7.85 (m, 7H, Ar-H), 11.44 (s,1H, OH, D_2_O exchangeable); Anal. Calcd for C_17_H_16_O_4_: C, 71.82; H, 5.67. Found: C, 72.00; H, 5.61.

*1-(4-Chlorophenyl)-3-(4-hydroxy-3-methoxyphenyl)prop-2-en-1-one* (**1c**). The chalcone was obtained in 77% yield; m.p.105 °C; IR (KBr, cm^−1^): 3433 (OH), 1664 (C=O), CH=CH (1614); ^1^H-NMR (DMSO-*d*_6_): δ 3.31 (s, 3H, OCH_3_), 6.61 (d, 1H, COCH=C*H*, *J* = 15 Hz), 7.28 (d, 1H, COC*H*=CH, *J* = 15 Hz), 7.33–7.87 (m, 7H, Ar-H), 11.44 (s,1H, OH, D_2_O exchangeable); Anal. Calcd for C_16_H_13_ClO_3_: C, 66.56; H, 4.54. Found: C, 66.41; H, 4.57.

*1-(4-Bromophenyl)-3-(4-hydroxy-3-methoxyphenyl) prop-2-en-1-one* (**1d**). The chalcone was obtained in 82% yield; m.p. 97 °C; IR (KBr, cm^−1^): 3429 (OH), 1672 (C=O), CH=CH (1606); ^1^H-NMR (DMSO-*d*_6_): δ 3.36 (s, 3H, OCH_3_), 6.89 (d, 1H, COCH=C*H*, *J* = 15 Hz), 7.22 (d, 1H, COC*H*=CH, *J* = 15 Hz), 7.30–7.74 (m, 7H, Ar-H), 11.22 (s, 1H, OH, D_2_O exchangeable); Anal. Calcd for C_16_H_13_BrO_3_: C, 57.68; H, 3.93. Found: C, 57.72; H, 3.82.

*3-(4-Hydroxy-3-methoxyphenyl)-1-(4-nitrophenyl) prop-2-en-1-one* (**1e**). The chalcone was obtained in 85% yield; m.p. 95 °C; IR (KBr, cm^−1^): 3424 (OH), 1684 (C=O), CH=CH (1618); ^1^H-NMR (DMSO-*d*_6_): δ 3.30 (s, 3H, OCH_3_), 6.89 (d, 1H, COCH=C*H*, *J* = 15 Hz), 7.29 (d, 1H, COC*H*=CH, *J* = 15 Hz), 7.36–7.97 (m, 7H, Ar-H), 11.72 (s, 1H, OH, D_2_O exchangeable); Anal. Calcd. for C_16_H_13_NO_5_: C, 64.21; H, 4.38; N, 4.68. Found: C, 64.19; H, 4.31; N, 4.69.

#### 3.1.2. General Procedure for the Preparation of **2a**–**e**

A solution of chalcone **1a**–**e** (10 mmol) in ethanol (95%, 30 mL) was refluxed with the appropriate amount of *p*-sulphamylphenylhydrazine (10 mmol) in glacial acetic acid (2 mL) for six hours. The reaction mixture was poured into crushed ice and kept overnight at room temperature. The separated crude solid was filtered off, washed successively with water, dried and crystallized from ethanol (95%) to give **2a**–**e** as needles.

*4-(2-(3-(4-Hydroxy-3-methoxyphenyl)-1-phenylallylidene)hydrazinyl)benzenesulfonamide* (**2a**). The hydrazone was obtained in 67% yield; m.p. 147 °C; IR (KBr, cm^−1^): 3430 (OH), 3391, 3322 (NH_2_, NH), 1632 (C=N), 1603 (CH=CH); ^1^H-NMR (DMSO-*d*_6_): δ 3.33 (s, 3H, OCH_3_), 6.85 (d, 1H, N=C−CH=C*H*, *J* = 13 Hz), 7.54 (d, 1H, N=C−C*H*=CH, *J* = 13 Hz), 7.33–7.82 (m, 12H, Ar-H), 8.21 (s, 1H, NH, D_2_O exchangeable), 9.23 (s, 2H, NH_2_, D_2_O exchangeable), 11.22 (s,1H, OH, D_2_O exchangeable). Anal. Calcd. for C_22_H_21_N_3_O_4_S: C, 62.40; H, 5.00; N, 9.92. Found: C, 62.47; H, 4.89; N, 9.88.

*4-(2-(3-(4-Hydroxy-3-methoxyphenyl)-1-(4-methoxyphenylallylidene)hydrazinyl)benzenesulfonamide* (**2b**). The hydrazone was obtained in 90% yield; m.p. 160 °C; IR (KBr, cm^−1^): 3432 (OH), 3389, 3320 (NH_2_, NH), 1629 (C=N), 1609 (CH=CH); ^1^H-NMR (DMSO-*d*_6_): δ 3.22 (s, 3H, OCH_3_), 3.26 (s, 3H, OCH_3_), 6.83 (d, 1H, N=C−CH=C*H*, *J* = 13 Hz), 7.53 (d, 1H, N=C−C*H*=CH, *J* = 13 Hz), 7.31–7.81 (m, 11H, Ar-H), 8.46 (s, 1H, NH, D_2_O exchangeable), 9.29 (s, 2H, NH_2_, D_2_O exchangeable), 11.31 (s, 1H, OH, D_2_O exchangeable). Anal. Calcd. for C_23_H_23_N_3_O_5_S: C, 60.91; H, 5.11; N, 9.27. Found: C, 60.87; H, 5.08; N, 9.23.

*4-(2-(1-(4-Chlorophenyl)-3-(4-hydroxy-3-methoxyphenylallylidene)hydrazinyl)benzenesulfonamide* (**2c**). The hydrazone was obtained in 87% yield; m.p. 103 °C; IR (KBr, cm^−1^): 3439 (OH), 3382, 3313 (NH_2_, NH), 1626 (C=N), 1612 (CH=CH); ^1^H-NMR (DMSO-*d*_6_): δ 3.22 (s, 3H, OCH_3_), 6.69 (d, 1H, N=C−CH=C*H*, *J* = 13 Hz), 7.56 (d, 1H, N=C−C*H*=CH, *J* = 13 Hz), 7.36–7.61 (m, 11H, Ar-H), 8.54 (s, 1H, NH, D_2_O exchangeable), 9.31 (s, 2H, NH_2_, D_2_O exchangeable), 11.33 (s, 1H, OH, D_2_O exchangeable). Anal. Calcd. for C_22_H_20_ClN_3_O_4_S: C, 57.70; H, 4.40; N, 9.18. Found: C, 57.76; H, 4.35; N, 9.22.

*4-(2-(1-(4-Bromophenyl)-3-(4-hydroxy-3-methoxyphenylallylidene)hydrazinyl)benzenesulfonamide* (**2d**). The hydrazone was obtained in 93% yield; m.p. 110 °C; IR (KBr, cm^−1^): 3435 (OH), 3383, 3314 (NH_2_, NH), 1633 (C=N), 1604 (CH=CH); ^1^H-NMR (DMSO-*d*_6_): δ 3.19 (s, 3H, OCH_3_), 6.81 (d, 1H, N=C−CH=C*H*, *J* = 13 Hz), 7.54 (d, 1H, N=C−C*H*=CH, *J* = 13 Hz), 7.41–7.71 (m, 11H, Ar-H), 8.61 (s, 1H, NH, D_2_O exchangeable), 9.39 (s, 2H, NH_2_, D_2_O exchangeable), 11.39 (s, 1H, OH, D_2_O exchangeable). Anal. Calcd. for C_22_H_20_BrN_3_O_4_S: C, 52.60; H, 4.01; N, 8.36. Found: C, 52.61; H, 3.89; N, 8.42.

*4-(2-(3-(4-Hydroxy-3-methoxyphenyl)-1-(4-nitrophenylallylidene)hydrazinyl)benzenesulfonamide* (**2e**). The hydrazone was obtained in 79% yield; m.p. 135 °C; IR (KBr, cm^−1^): 3441 (OH), 3379, 3310 (NH_2_, NH), 1627 (C=N), 1619 (CH=CH); ^1^H-NMR (DMSO-*d*_6_): δ 3.37 (s, 3H, OCH_3_), 6.79 (d, 1H, N=C−CH=C*H*, *J* = 13 Hz), 7.61 (d, 1H, N=C−C*H*=CH, *J* = 13 Hz), 7.26–7.76 (m, 11H, Ar-H), 8.73 (s, 1H, NH, D_2_O exchangeable), 9.40 (s, 2H, NH_2_, D_2_O exchangeable), 11.38 (s, 1H, OH, D_2_O exchangeable). Anal. Calcd. for C_22_H_20_N_4_O_6_S: C, 56.40; H, 4.30; N, 11.96. Found: C, 56.39; H, 4.31; N, 11.95.

#### 3.1.3. General Procedure for the Preparation of **3a**–**e**

A mixture of the appropriate hydrazone **2a**–**e** (10 mmol) and acetic anhydride (15 mL) was heated under reflux for three hours. After the reaction mixture attained room temperature, it was poured into crushed ice and the oily product deposited was decanted from water and extracted with ether. The ether layer was washed three times with water, dried over anhydrous sodium sulphate and evaporated. The precipitate obtained was crystallized from ethanol (95%) to afford the corresponding pyrazoles **3a**–**e** as needles.

*2-Methoxy-4-(3-phenyl-1-(4-sulfamoylphenyl)-1H-pyrazol-5-yl)phenyl acetate* (**3a**). The pyrazole was obtained in 69% yield; m.p. 187 °C; IR (KBr, cm^−1^): 3386 (NH_2_), 1750 (C=O), 1632 (C=N); ^1^H-NMR (DMSO-*d*_6_): δ 2.39 (s, 3H, CH_3_), 3.33 (s, 3H, OCH_3_), 6.72 (s, 1H, pyrazole C_4_-H), 7.24–8.29 (m, 12H, Ar-H), 9.33 (s, 2H, NH_2_, D_2_O exchangeable). Anal. Calcd. for C_24_H_21_N_3_O_5_S: C, 62.19; H, 4.57; N, 9.07. Found: C, 62.19; H, 4.40; N, 9.09.

*2-Methoxy-4-(3-(4-methoxyphenyl)-1-(4-sulfamoylphenyl)-1H-pyrazol-5-yl)phenyl acetate* (**3b**). The pyrazole was obtained in 91% yield; m.p. 193 °C; IR (KBr, cm^−1^): 3384 (NH_2_), 1751 (C=O), 1641 (C=N); ^1^H-NMR (DMSO-*d*_6_): δ 2.28 (s, 3H, CH_3_), 3.21 (s, 3H, OCH_3_), 3.34 (s, 3H, OCH_3_), 6.87 (s, 1H, pyrazole C_4_-H), 7.35–7.94 (m, 11H, Ar-H), 9.76 (s, 2H, NH_2_, D_2_O exchangeable). Anal. Calcd. for C_25_H_23_N_3_O_6_S: C, 60.84; H, 4.70; N, 8.51. Found: C, 60.75; H, 4. 68; N, 8.59.

*4-(3-(4-Chlorophenyl)-1-(4-sulfamoylphenyl)-1H-pyrazol-5-yl)-2-methoxyphenyl acetate* (**3c**).The pyrazole was obtained in 76% yield; m.p. 172 °C; IR (KBr, cm^−1^): 3379 (NH_2_), 1748 (C=O), 1644 (C=N); ^1^H-NMR (DMSO-*d*_6_): δ 2.22 (s, 3H, CH_3_), 3.27 (s, 3H, OCH_3_), 6.83 (s, 1H, pyrazole C_4_-H), 7.29–7.86 (m, 11H, Ar-H), 9.92 (s, 2H, NH_2_, D_2_O exchangeable). Anal. Calcd. for C_24_H_20_ClN_3_O_5_S: C, 57.89; H, 4.05; N, 8.44. Found: C, 57.80; H, 4.10; N, 8.40.

*4-(3-(4-Bromophenyl)-1-(4-sulfamoylphenyl)-1H-pyrazol-5-yl)-2-methoxyphenyl acetate* (**3d**). The pyrazole was obtained in 78% yield; m.p. 177 °C; IR (KBr, cm^−1^): 3391 (NH_2_), 1746 (C=O), 1654 (C=N); ^1^H-NMR (CDCl_3_): δ 2.30 (s, 3H, CH_3_), 3.31 (s, 3H, OCH_3_), 6.85 (s, 1H, pyrazole C_4_-H), 7.13–7.64 (m, 11H, Ar-H), 9.71 (s, 2H, NH_2_, D_2_O exchangeable); ^13^C-NMR (CDCl_3_): δ 20.7 (CH_3_), 56.0 (OCH_3_), 111.9, 121.5, 121.8, 123.4, 128.0, 130.1, 132.0, 133.7, 136.9, 140.4, 141.8, 142.2, 144.8, 151.5 (pyrazole, C_6_H_3_ and 2 C_6_H_4_ C), 168.8 (C=N) and 189.3 (C=O). Anal. Calcd. for C_24_H_20_BrN_3_O_5_S: C, 53.14; H, 3.72; N, 7.75. Found: C, 53.10; H, 3.66; N, 7.80.

*2-Methoxy-4-(3-(4-nitrophenyl)-1-(4-sulfamoylphenyl)-1H-pyrazol-5-yl)phenyl acetate* (**3e**). The pyrazole was obtained in 69% yield; m.p.197 °C; IR (KBr, cm^−1^): 3393 (NH_2_), 1752 (C=O), 1656 (C=N); ^1^H-NMR (DMSO-*d*_6_): δ 2.26 (s, 3H, CH_3_), 3.33 (s, 3H, OCH_3_), 6.93 (s, 1H, pyrazole C_4_-H), 7.29–8.10 (m, 11H, Ar-H), 10.37 (s, 2H, NH_2_, D_2_O exchangeable). Anal*.* Calcd. for C_24_H_20_N_4_O_7_S: C, 56.69; H, 3.96; N, 11.02. Found: C, 56.72; H, 3.95; N, 11.10.

#### 3.1.4. General Procedure for the Preparation of **4a**–**e**

A mixture of the appropriate chalcone **1a**–**e** (10 mmol) in ethanol (30 mL) was heated under reflux with thiosemicarbazide (12 mmol) in glacial acetic acid (2 mL) for 7 h, then the reaction mixture was poured into crushed ice and kept overnight at room temperature. The separated crude solid was filtered off, washed successively with water, dried and crystallized from ethanol/chloroform to give **4a**–**e** as needles.

*5-(4-Hydroxy-3-methoxyphenyl)-3-phenyl-4*,*5-dihydro-1H-pyrazole-1-carbothioamide* (**4a**). The 4,5-dihydropyrazole was obtained in 81% yield; m.p. 149 °C; IR (KBr, cm^−1^): 3430 (OH), 3394 (NH_2_), 1647 (C=N), 1246 (C=S); ^1^H-NMR (DMSO-*d*_6_): δ 3.11 (dd, 1H, pyrazolyl-H_A_, *J*_AX_ = 3.6 Hz, *J*_AB_ = 16 Hz), 3.31 (s, 3H, OCH_3_), 3.72 (dd, 1H, pyrazolyl-H_B_, *J*_BX_ = 12 Hz, *J*_AB_ = 16 Hz), 5.39 (dd, 1H, pyrazolyl-H_X_, *J*_AX_ = 3.6 Hz, *J*_BX_ = 12 Hz), 7.26–7.82 (m, 8H, Ar-H), 10.73 (s, 2H, NH_2_, D_2_O exchangeable), 11.21 (s, 1H, OH, D_2_O exchangeable). Anal. Calcd. for C_17_H_17_N_3_O_2_S: C, 62.36; H, 5.23; N, 12.83. Found: C, 62.33; H, 5.20; N, 12.88.

*5-(4-Hydroxy-3-methoxyphenyl)-3-(4-methoxyphenyl)-4*,*5-dihydro-1H-pyrazole-1-carbothioamide* (**4b**). The 4,5-dihydropyrazole was obtained in 83% yield; m.p. 156 °C; IR (KBr, cm^−1^): 3432 (OH), 3390 (NH_2_), 1650 (C=N), 1245 (C=S); ^1^H-NMR (DMSO-*d*_6_): δ 3.20 (dd, 1H, pyrazolyl-H_A_, *J*_AX_ = 3.6 Hz, *J*_AB_ = 16 Hz), 3.32 (s, 3H, OCH_3_), 3.39 (s, 3H, OCH_3_), 3.75 (dd, 1H, pyrazolyl-H_B_, *J*_BX_ = 12 Hz, *J*_AB_ = 16 Hz), 5.39 (dd, 1H, pyrazolyl-H_X_, *J*_AX_ = 3.6 Hz, *J*_BX_ = 12 Hz), 7.27–7.77 (m, 7H, Ar-H), 10.73 (s, 2H, NH_2_, D_2_O exchangeable), 11.30 (s, 1H, OH, D_2_O exchangeable); ^13^C-NMR (DMSO-*d*_6_): δ 48.2, 70.6 (C_3_ and C_4_ dihydropyrazole), 56.2, 56.5 (2 OCH_3_), 109.7, 113.2, 115.7, 119.4, 122.9, 126.0, 134.3, 143.4, 148.6, 149.2 (C_6_H_3_ and C_6_H_4_ C), 169.4 (C=N) and 177.8 (C=S). Anal. Calcd. for C_18_H_19_N_3_O_3_S: C, 60.49; H, 5.36; N, 11.76. Found: C, 60.50; H, 5.34; N, 11.81.

*3-(4-Chlorophenyl)-5-(4-hydroxy-3-methoxyphenyl)-4*,*5-dihydro-1H-pyrazole-1-carbothioamide* (**4c**). The 4,5-dihydropyrazole was obtained in 87% yield; m.p. 172 °C; IR (KBr, cm^−1^): 3439 (OH), 3390 (NH_2_), 1652 (C=N), 1248 (C=S); ^1^H-NMR (DMSO-*d*_6_): δ 3.08 (dd, 1H, pyrazolyl-H_A_, *J*_AX_ = 3.6 Hz, *J*_AB_ = 16 Hz), 3.27 (s, 3H, OCH_3_), 3.79 (dd, 1H, pyrazolyl-H_B_, *J*_BX_ = 12 Hz, *J*_AB_ = 16 Hz), 5.44 (dd, 1H, pyrazolyl-H_X_, *J*_AX_ = 3.6 Hz, *J*_BX_ = 12 Hz), 7.29–8.01 (m, 7H, Ar-H), 10.79 (s, 2H, NH_2_, D_2_O exchangeable), 11.33 (s, 1H, OH, D_2_O exchangeable). Anal. Calcd. for C_17_H_16_ClN_3_O_2_S: C, 56.43; H, 4.46; N, 11.61. Found: C, 56.33; H, 4.42; N, 11.71.

*3-(4-Bromophenyl)-5-(4-hydroxy-3-methoxyphenyl)-4*,*5-dihydro-1H-pyrazole-1-carbothioamide* (**4d**). The 4,5-dihydropyrazole was obtained in 79% yield; m.p. 146 °C; IR (KBr, cm^−1^): 3440 (OH), 3393 (NH_2_), 1657 (C=N), 1247 (C=S); ^1^H-NMR (DMSO-*d*_6_): δ 3.12 (dd, 1H, pyrazolyl-H_A_, *J*_AX_ = 3.6 Hz, *J*_AB_ = 16 Hz), 3.29 (s, 3H, OCH_3_), 3.76 (dd, 1H, pyrazolyl-H_B_, *J*_BX_ = 12 Hz, *J*_AB_ = 16 Hz), 5.46 (dd, 1H, pyrazolyl-H_X_, *J*_AX_ = 3.6 Hz, *J*_BX_ = 12 Hz), 7.23–7.61 (m, 7H, Ar-H), 10.93 (s, 2H, NH_2_, D_2_O exchangeable), 11.27 (s, 1H, OH, D_2_O exchangeable). Anal. Calcd. for C_17_H_16_BrN_3_O_2_S: C, 50.25; H, 3.97; N, 10.34. Found: C, 50.23; H, 3.89; N, 10.37.

*5-(4-Hydroxy-3-methoxyphenyl)-3-(4-nitrophenyl)-4*,*5-dihydro-1H-pyrazole-1-carbothioamide* (**4e**). The 4,5-dihydropyrazole was obtained in 93% yield; m.p.: 166 °C; IR (KBr, cm^−1^): 3451 (OH), 3387 (NH_2_), 1646 (C=N), 1232 (C=S); ^1^H-NMR (DMSO-*d*_6_): δ 3.07 (dd, 1H, pyrazolyl-H_A_, *J*_AX_ = 3.6 Hz, *J*_AB_ = 16 Hz), 3.32 (s, 3H, OCH_3_), 3.74 (dd, 1H, pyrazolyl-H_B_, *J*_BX_ = 12 Hz, *J*_AB_ = 16 Hz), 3.39 (dd, 1H, pyrazolyl-H_X_, *J*_AX_ = 3.6 Hz, *J*_BX_ = 12 Hz), 7.27–7.99 (m, 7H, Ar-H), 10.71 (s, 2H, NH_2_, D_2_O exchangeable), 11.24 (s, 1H, OH, D_2_O exchangeable). Anal. Calcd. for C_17_H_16_N_4_O_4_S: C, 54.83; H, 4.33; N, 15.04. Found: C, 54.87; H, 4.33; N, 15.08.

#### 3.1.5. General Procedure for the Preparation of **5a**–**e**

A mixture of the appropriate chalcone **1a**–**e** (10 mmol) in ethanol (95%) (30 mL) was heated under reflux with isonicotinic acid hydrazide (10 mmol) in glacial acetic acid (2 mL) for five hours. The reaction mixture was treated as mentioned for the preparation of **4a**–**e** to give the corresponding 4,5-dihydropyrazoles **5a**–**e**.

*(5-(4-Hydroxy-3-methoxyphenyl)-3-phenyl-4*,*5-dihydro-1H-pyrazol-1-yl)(pyridin-4-yl)methanone* (**5a**). The corresponding 4,5-dihydropyrazole was obtained in 81% yield; m.p. 159 °C; IR (KBr, cm^−1^): 3472 (OH), 1645 (C=N), 1630 (C=O); ^1^H-NMR (DMSO-*d*_6_): δ 3.14 (dd, 1H, pyrazolyl-H_A_, *J*_AX_ = 3.6 Hz, *J*_AB_ = 16 Hz), 3.32 (s, 3H, OCH_3_), 3.84 (dd, 1H, pyrazolyl-H_B_, *J*_BX_ = 12 Hz, *J*_AB_ = 16 Hz), 5.40 (dd, 1H, pyrazolyl-H_X_, *J*_AX_ = 3.6 Hz, *J*_BX_ = 12 Hz), 6.81–7.48 (m, 12H, Ar-H), 11.27 (s, 1H, OH, D_2_O exchangeable). Anal. Calcd. for C_22_H_19_N_3_O_3_: C, 70.76; H, 5.13; N, 11.25. Found: C, 70.77; H, 5.12; N, 11.27.

*(5-(4-Hydroxy-3-methoxyphenyl)-3-(4-methoxyphenyl)-4*,*5-dihydro-1H-pyrazol-1-yl)(pyridin-4-yl)methanone* (**5b**). The corresponding 4,5-dihydropyrazole was obtained in 69% yield; m.p. 166 °C; IR (KBr, cm^−1^): 3492 (OH), 1649 (C=N), 1628 (C=O); ^1^H-NMR (DMSO-*d*_6_): δ 3.16 (dd, 1H, pyrazolyl-H_A_, *J*_AX_ = 3.6 Hz, *J*_AB_ = 16 Hz), 3.25 (s, 3H, OCH_3_), 3.31 (s, 3H, OCH_3_), 3.75 (dd, 1H, pyrazolyl-H_B_, *J*_BX_ = 12 Hz, *J*_AB_ = 16 Hz), 5.37 (dd, 1H, pyrazolyl-H_X_, *J*_AX_ = 3.6 Hz, *J*_BX_ = 12 Hz), 6.79–7.49 (m, 11H, Ar-H), 11.31 (s, 1H, OH, D_2_O exchangeable). Anal. Calcd. for C_23_H_21_N_3_O_4_: C, 68.47; H, 5.25; N, 10.42. Found: C, 68.51; H, 5.26; N, 10.48.

*(3-(4-Chlorophenyl)-5-(4-hydroxy-3-methoxyphenyl)-4*,*5-dihydro-1H-pyrazol-1-yl)(pyridin-4-yl)methanone* (**5c**). The corresponding 4,5-dihydropyrazole was obtained in 89% yield; m.p. 142 °C; IR (KBr, cm^−1^): 3481 (OH), 1655 (C=N), 1631 (C=O); ^1^H-NMR (DMSO-*d*_6_): δ 3.18 (dd, 1H, pyrazolyl-H_A_, *J*_AX_ = 3.6 Hz, *J*_AB_ = 16 Hz), 3.27 (s, 3H, OCH_3_), 3.80 (dd, 1H, pyrazolyl-H_B_, *J*_BX_ = 12 Hz, *J*_AB_ = 16 Hz), 5.46 (dd, 1H, pyrazolyl-H_X_, *J*_AX_ = 3.6 Hz, *J*_BX_ = 12 Hz), 6.83–7.51 (m, 11H, Ar-H), 11.22 (s, 1H, OH, D_2_O exchangeable); ^13^C-NMR (DMSO-*d*_6_): δ 40.3, 66.8 (C_3_ and C_4_ dihydropyrazole), 56.3 (OCH_3_), 110.6, 116.1, 118.7, 123.5, 124.8, 129.1, 130.8, 137.1 138.3, 146.0, 146.5, 148.5, 150.2 (C_6_H_3_, C_6_H_4_ and pyridine C), 164.3 (C=N) and 188.4 (C=O) . Anal. Calcd. for C_22_H_18_ClN_3_O_3_: C, 64.79; H, 4.45; N, 10.30. Found: C, 64.69; H, 4.45; N, 10.29.

*(3-(4-Bromophenyl)-5-(4-hydroxy-3-methoxyphenyl)-4*,*5-dihydro-1H-pyrazol-1-yl)(pyridin-4-yl)methanone* (**5d**). The 4,5-dihydropyrazole was obtained in 86% yield; m.p. 168 °C; IR (KBr, cm^−1^): 3466 (OH), 1654 (C=N), 1633 (C=O); ^1^H-NMR (DMSO-*d*_6_): δ 3.20 (dd, 1H, pyrazolyl-H_A_, *J*_AX_ = 3.6 Hz, *J*_AB_ = 16 Hz), 3.22 (s, 3H, OCH_3_), 3.77 (dd, 1H, pyrazolyl-H_B_, *J*_BX_ = 12 Hz, *J*_AB_ = 16 Hz), 5.47 (dd, 1H, pyrazolyl-H_X_, *J*_AX_ = 3.6 Hz, *J*_BX_ = 12 Hz), 6.82–7.52 (m, 11H, Ar-H), 11.20 (s, 1H, OH, D_2_O exchangeable). Anal. Calcd. for C_22_H_18_BrN_3_O_3_: C, 58.42; H, 4.01; N, 9.29. Found: C, 58.31; H, 3.89; N, 9.28.

*(5-(4-Hydroxy-3-methoxyphenyl)-3-(4-nitrophenyl)-4*,*5-dihydro-1H-pyrazol-1-yl)(pyridin-4-yl) methanone* (**5e**). The 4,5-dihydropyrazole was obtained in 97% yield; m.p. 171 °C; IR (KBr, cm^−1^): 3471 (OH), 1649 (C=N), 1634 (C=O); ^1^H-NMR (DMSO-*d*_6_): δ 3.17 (dd, 1H, pyrazolyl-H_A_, *J*_AX_ = 3.6 Hz, *J*_AB_ = 16 Hz), 3.39 (s, 3H, OCH_3_), 3.69 (dd, 1H, pyrazolyl-H_B_, *J*_BX_ = 12 Hz, *J*_AB_ = 16 Hz), 5.42 (dd, 1H, pyrazolyl-H_X_, *J*_AX_ = 3.6 Hz, *J*_BX_ = 12 Hz), 6.84–7.71 (m, 11H, Ar-H), 11.89 (s, 1H, OH, D_2_O exchangeable). Anal. Calcd. for C_22_H_18_N_4_O_5_: C, 63.15; H, 4.34; N, 13.39. Found: C, 63.14; H, 4.28; N, 13.47.

### 3.2. Determination of Antimicrobial Activity

All compounds were tested against four different microorganisms *Staphylococcus aureus*, *Escherichia coli*, *Pseudomonas aeruginosa*, *Candida albicans*. The agar well-diffusion method was applied for the determination of inhibition zone and minimum inhibitory concentration (MIC). Briefly, 0.75 mL of broth culture containing *ca.* 10^6^ colony-forming units (CFU) per mL of the test strain was added to 75 mL of nutrient agar medium at 45 °C, mixed well, and then poured into a 15 cm sterile metallic Petri plate. The medium was allowed to solidify, and 8 mm wells were dug with a sterile metallic borer. Then, a DMSO solution of the test sample (1 mL) at 1 mg/mL was added to the respective wells. DMSO served as negative control, and the standard antimicrobial drugs Rifampicin (5 μg/disc) and Ampicillin (10 μg/disc) were used as positive controls. Triplicate plates for each microorganism strain were prepared and were incubated aerobically at 37 °C for 24 h. The activity was determined by measuring the diameter of zone showing complete inhibition (mm), thereby, the zones were precisely measured with the aid of a Vernier caliper (precision 0.1 mm). The growth inhibition was calculated with reference to the positive control. For the individual compounds that showed inhibition zones >10 mm, MIC values were determined by means of the agar well-diffusion method for concentrations of 1.0, 0.50, 0.25, 0.125, 0.063 and 0.031 mg/mL in DMSO. The tests were performed in triplicate, and the results were averaged. Also minimum bactericidal concentrations (MBC) were determined for all chloro derivatives which exhibited high activities (compounds **1c**, **2c**, **4c** and **5c**) for concentrations of 1.0, 0.50, 0.25, 0.125, 0.063 and 0.031 mg/mL in DMSO. All our results are listed in Table 1 and Table 2.

### 3.3. DPPH Based Free Radical Scavenging Activity

Since DPPH is a stable free radical containing an odd electron in its structure, it is usually utilized for detection of the radical scavenging activity. Aliquots of different concentrations (20–100 µg/mL) of the test sample is added to 100 µL solution DPPH (4 mg/100 mL methanol). Absorbance at 517 nm is determined after 30 min. Each experiment was done in triplicate and average is taken. Vitamin C was used as a positive control and percentage of free radical scavenging was expressed as inhibition from the given formula:

(1) $$\text{\% inhibition of DPPH radical}=\frac{\text{Abs. of control}-\text{Abs. of sample}}{\text{Abs. of control}}\times 100$$

Efficacy was calculated for 0.25 mg/mL of each compound by using the following equation:

(2) $$\text{Efficacy}=\frac{\text{DPPH scavenging \% of compound at 0.25 mL}}{\text{DPPH scavenging \% of vitamin C at 0.25 mL}}$$

Calculated antioxidant data of all the tested samples were summarized in Table 3.

### 3.4. Nitric Oxide Radical Scavenging Activity

Nitric oxide was generated from sodium nitroprusside and measured by Griess’ reaction [62,63]. Reagents are sodium nitroprusside (10 mM), phosphate buffer saline and Griess reagent (1 g of sulphanilic acid + 0.1 g naphthylethylene diamine dihydrochloride). 20 µL sodium nitroprusside, 5 µL phosphate buffer and 5 µL of compound were incubated at 25 °C for 2.30 h. After incubation, 20 µL of griess reagent was added to the previous mixture and allowed to stand for 30 min. The absorbance of the colour developed during diazotization of nitrite with sulphanilamide and its subsequent coupling with napthylethylenediamine hydrochloride was observed at 550 nm on spectrophotometer. Each experiment was done in triplicate and average is taken. Vitamin C was used as positive control and percentage of free radical scavenging was expressed as inhibition from the formula:

(3) $$\text{\% inhibition of NO radical}=\frac{\text{Abs. of control}-\text{Abs. of sample}}{\text{Abs. of control}}\times 100$$

Efficacy was calculated for 0.25 mg/mL of each compound by using the following equations:

(4) $$\text{Efficacy}=\frac{\text{NO scavenging \% of compound at 0.25 mL}}{\text{NO scavenging \% of vitamin C at 0.25 mL}}$$

Calculated anti-inflammatory data of all the tested samples were summarized in (Table 4).

## 4. Conclusions

This work demonstrates a rapid, efficient method for the synthesis of new pyrazole and dihydropyrazole derivatives. All synthesized compounds were characterized by spectral data (IR, ^1^H-NMR and ^13^C-NMR) and the structures were consistent with the data. All the synthesized compounds were tested for their antimicrobial activity against four test organisms. The results showed that the compounds that having pharmacophores with lipophilic properties such as chloro and bromo substituents exhibited the greatest antimicrobial activities. Also two pharmacological activities namely antioxidant and anti-inflammatory activity, were tested for the chloro derivatives **1c**, **2c**, **3c**, **4c**, **5c**. These activities vary according to their structures and functional groups.
